# NADP^+^-Dependent Dehydrogenase *SCO3486* and Cycloisomerase *SCO3480*: Key Enzymes for 3,6-Anhydro-L-Galactose Catabolism in *Streptomyces coelicolor* A3(2)

**DOI:** 10.4014/jmb.2103.03030

**Published:** 2021-04-06

**Authors:** Maral Tsevelkhorloo, Sang Hoon Kim, Dae-Kyung Kang, Chang-Ro Lee, Soon-Kwang Hong

**Affiliations:** 1 Department of Biosciences and Bioinformatics, Myongji University, Yongin 17058, Republic of Korea; 2 Department of Animal Resources Science, Dankook University, Cheonan 31116, Republic of Korea

**Keywords:** *Streptomyces coelicolor* A3 (2), 3,6-anhydro-L-galactose catabolism, 3,6-anhydro-L-galactose dehydrogenase, 3,6-anhydro-L-galactonate cycloisomerase, *SCO3480*, *SCO3486*

## Abstract

Agarose is a linear polysaccharide composed of D-galactose and 3,6-anhydro-L-galactose (AHG). It is a major component of the red algal cell wall and is gaining attention as an abundant marine biomass. However, the inability to ferment AHG is considered an obstacle in the large-scale use of agarose and could be addressed by understanding AHG catabolism in agarolytic microorganisms. Since AHG catabolism was uniquely confirmed in *Vibrio* sp. EJY3, a gram-negative marine bacterial species, we investigated AHG metabolism in *Streptomyces coelicolor* A3(2), an agarolytic gram-positive soil bacterium. Based on genomic data, the *SCO3486* protein (492 amino acids) and the *SCO3480* protein (361 amino acids) of *S. coelicolor* A3(2) showed identity with H2IFE7.1 (40% identity) encoding AHG dehydrogenase and H2IFX0.1 (42% identity) encoding 3,6-anhydro-L-galactonate cycloisomerase, respectively, which are involved in the initial catabolism of AHG in *Vibrio* sp. EJY3. Thin layer chromatography and mass spectrometry of the bioconversion products catalyzed by recombinant *SCO3486* and *SCO3480* proteins, revealed that *SCO3486* is an AHG dehydrogenase that oxidizes AHG to 3,6-anhydro-L-galactonate, and *SCO3480* is a 3,6-anhydro-L-galactonate cycloisomerase that converts 3,6-anhydro-L-galactonate to 2-keto-3-deoxygalactonate. *SCO3486* showed maximum activity at pH 6.0 at 50°C, increased activity in the presence of iron ions, and activity against various aldehyde substrates, which is quite distinct from AHG-specific H2IFE7.1 in *Vibrio* sp. EJY3. Therefore, the catabolic pathway of AHG seems to be similar in most agar-degrading microorganisms, but the enzymes involved appear to be very diverse.

## Introduction

Marine red algal cells contain high amounts of cellular carbohydrates and low amounts of lignin, and thus have gained attention as a sustainable next-generation biomass resource. Red algae-derived carbohydrates can replace fossil fuels, which are expected to be depleted in the near future, and can be converted into chemical feedstocks and bioenergy [1, 2].

Consisting mainly of carbohydrates that make up the majority of red algal cells, agar is a copolymer of the agarobiose unit (4‐O‐β‐D‐galactopyranosyl‐3,6‐anhydro‐L‐galactose) and the porphyrobiose unit (4-O-β-D-galactopyranosyl-L‐galactose-6-sulfate), linked by alpha-1,3 glycosidic bonds. The composition ratio of the two units varies depending on the species of red algae. Agar is called ‘agarose’ when agarobiose is the main component, and ‘porphyran’ when porphyrobiose is the main component [1, 3].

Agar has been widely used as a food, food additive, culturing medium, and support material for techniques like electrophoresis and chromatography [2]. Moreover, as the various biological activities of agar-derived oligosaccharides and agar decomposition products are discovered, the industrial use of agar is expected to expand [4]. Agarose, in nature, is mainly hydrolyzed by microbial beta-agarases into neoagarobiose (3,6-anhydro-L-galactosyl-α-1,3-D-galactose), which is further hydrolyzed by neoagarooligosaccharide (NAOS) hydrolase into monomers D‐galactose (D‐Gal) and 3,6‐anhydro‐L‐galactose (L-AHG) [5]. D‐Gal is a valuable chemical feedstock that can be converted into bioenergy or other useful chemicals [4]. Additionally, L-AHG is a rare monosaccharide found only in red algae, and has been reported to have antioxidant activity as well as the ability to suppress dental caries [6, 7].

Despite the benefits of red algae, such as being a rich source of carbohydrates, the fact that L-AHG is not available as a carbon source for industrial fermentative microorganisms imposes serious restrictions on the use of red algal biomass. Because it accounts for up to 34% of the dry weight of red algae, the bioconversion of L-AHG into a fermentable form is an important issue in need of early resolution to increase the economic value of red algal biomass [8]. Therefore, if the catalytic pathway of L-AHG conversion in agar-degrading microorganisms is identified, L-AHG can be converted into fermentable sugars and used by industrial fermentative microorganisms through metabolic engineering, as demonstrated in the case of ethanol production in engineered ethanologenic *Escherichia coli* [9].

The L-AHG metabolic pathway was recently documented for the first time, as a new catabolic pathway that uses L-AHG as the only carbon source in an agar-degrading, gram-negative marine bacterium, *Vibrio* sp. EJY3 [9]. L-AHG undergoes a continuous reaction catalyzed by two major enzymes: AHG dehydrogenase (E.C. 1.2.1.92) and 3,6-anhydro-L-galactonate (AHGA) cycloisomerase (E.C. 5.5.1.25). AHG dehydrogenase oxidizes L-AHG to AHGA, and requires NADP^+^ as a cofactor. The resulting AHGA is converted to 2-keto-3-deoxygalactonate (KDGal) by AHGA cycloisomerase, and KDGal is finally metabolized after entering the central metabolic pathway [10].

**Fig. 1:**
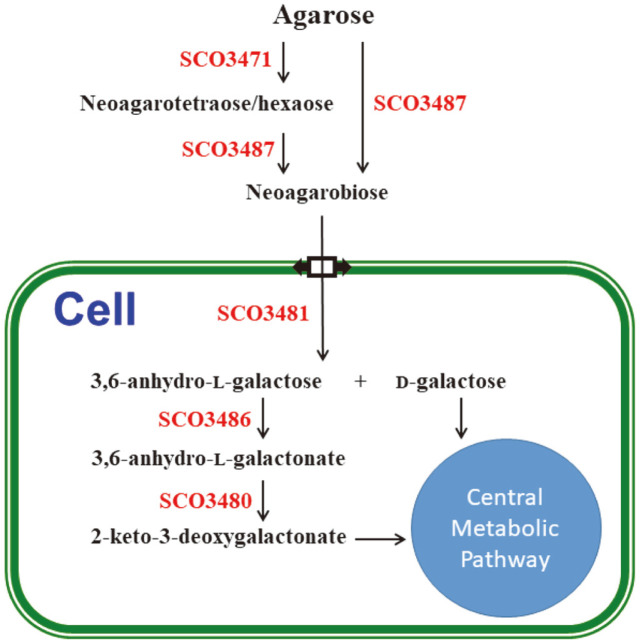
A proposed agar hydrolytic pathway in *Streptomyces coelicolor* A3(2). Overall enzymology was constructed from the enzymatic properties validated and genetic information. The enzymatic properties of agarases, DagA (SCO3471), DagB (SCO3487), and DagC (SCO3481), are explained in the text. Two proteins, SCO3486 and SCO3480, studied in this article, are also presented in the pathway.

The gram-positive soil microorganism *Streptomyces coelicolor* A3(2) is the only bacterium in the genus *Streptomyces* that can grow using agar as the only carbon source [11]. Our previous studies on the agar decomposition system of *S. coelicolor* A3(2) demonstrated that the strain produced two types of beta-agarases, DagA [12] and DagB [13], and one type of NAOS hydrolase (SCO3481), which act in succession and decompose agar into monosaccharides D-Gal and L-AHG [1], as illustrated in Fig. 1. In this study, the catalytic fate of L-AHG was biochemically investigated in *S. coelicolor* A3(2).

## Materials and Methods

### Bacterial Strains and Culture Conditions

*Escherichia coli* ER2566 and plasmid pET-28a(+) were used for cloning and expression of the *SCO3486* and *SCO3480* genes, respectively. *E. coli* ER2566 was cultured in Luria-Bertani (LB) medium [14] at 37°C, unless otherwise specified. If necessary, kanamycin (50 μg/ml) was added. *S. coelicolor* A3(2) obtained from the John Innes Centre (United Kingdom) was cultured in R2YE medium and used for chromosomal DNA isolation, as described previously [15].

### Chemicals

Bacterial media and kanamycin were purchased from Duchefa Biochemie (Netherlands). *Pfu* polymerase was obtained from Enzynomics Co., Ltd. (Korea), and PCR primers were synthesized by Genotech (Korea). DNA-modifying enzymes and kits for recombinant DNA technology were purchased from Takara Bio (Japan). TALON metal affinity resin and silica gel plates (60G F_254_) for thin-layer chromatography were purchased from Clontech Laboratories Inc. (CA, USA) and Merck KGaA (Darmstadt, Germany), respectively. L-AHG was purchased from DyneBio Inc. (Korea). Other fine chemicals were purchased from Sigma-Aldrich Corporation (USA), unless otherwise specified.

### Cloning of the *SCO3486* and *SCO3480* Genes

*SCO3486* (NCBI Reference Sequence: NP_627689.1) and *SCO3480* (NP_627683.1) were annotated as putative aldehyde dehydrogenase and putative racemase, respectively. The 1,479-bp (*SCO3486*) and 1,086-bp (*SCO3480*) DNA fragments were amplified by PCR using the chromosomal DNA of *S. coelicolor* A3(2) as a template. A forward primer (5'-CGCGCGGCAGCCATATGACTCACGAACTCTTCGACAG-3'; NdeI site underlined) and a reverse primer (5'-GCTCGAATTCGGATCCTCAGACAGCGTGCCGCACGT-3'; BamHI site underlined), were designed for *SCO3486*, and a forward primer (5'-CGCGCGGCAGCCATATGATCGAACGGGTACGCACCGA-3'; NdeI site underlined) and a reverse primer (5'-GCTCGAATTCGGATCCTCACCCCACCGCCAGCCGGC-3'; BamHI site underlined) were designed for *SCO3480*. Amplification reactions were performed as previously described [12]. Each PCR product was digested with NdeI and BamHI restriction enzymes, and then ligated into pET-28a(+) treated with the same restriction enzymes to construct pHis-SCO3486 and pHis-SCO3480, respectively.

### Purification of Recombinant SCO3486 and SCO3480

*E. coli* ER2566/pHis-SCO3486 and *E. coli* ER2566/pHis-SCO3480 cells were cultivated in LB broth containing kanamycin, with shaking at 37°C. The expression of each recombinant protein was induced by adding 0.5 mM of isopropyl-β-D-thiogalactopyranoside (IPTG) at an optical density at 600 nm (OD_600_) of 0.5, followed by further cultivation at 16°C overnight. The cells were harvested by centrifugation for 10 min at 10,000 ×*g* and resuspended in a binding buffer (30 mM Tris-HCl, pH 7.9, 250 mM NaCl). Cells were then disrupted by sonication (output control 5 and duty cycle 50%) using a Branson Sonifier 450 (Branson Ultrasonics Corp., USA), followed by centrifugation at 15,000 ×*g* for 30 min at 4°C. His-tagged recombinant protein was purified from the supernatant by TALON metal affinity chromatography according to the supplier's instructions. The eluate with 200 mM imidazole was dialyzed against 30 mM Tris-HCl (pH 7.9) containing 100 mM NaCl overnight at 4°C to remove imidazole. The molecular weight and purity of the purified protein were confirmed by 0.1% sodium dodecyl sulfate-12% polyacrylamide gel electrophoresis (SDS-PAGE), as previously described [16]. Protein concentration was determined according to the method described by Bradford [17].

### Biochemical Characterization of SCO3486

According to a protein BLAST search, SCO3486 was expected to be an AHG dehydrogenase that oxidizes L-AHG to AHGA. This oxidative reaction is coupled with the reduction of NADP^+^ to NADPH, which can be measured using a spectrophotometer at 340 nm, as described previously [9]. Unless specified otherwise, the reaction mixture (50 μl) contained 10 μg L-AHG, 1.5 mM NADP^+^, and 15 μg SCO3486 in 50 mM sodium phosphate buffer (pH 6.0), and was incubated at 50°C for 10 min (hereafter referred to as the standard conditions). After heat treatment at 95°C for 5 min, an increase in absorbance at 340 nm (A_340_) was recorded using a Spectronic Unicam Genesys & Spectrophotometer (Thermo Scientific, USA). An extinction coefficient of 6,220 M^-1^ cm^-1^ was used to calculate the amount of NADH formed during the enzymatic reaction.

The pH profile of SCO3486 enzyme activity was measured in various pH ranges, specifically pH 4.0-5.0 (50 mM sodium citrate buffer), pH 6.0-7.0 (50 mM sodium phosphate buffer), pH 8.0-9.0 (50 mM Tris-HCl buffer), and pH 10.0 (50 mM glycine-NaOH buffer), at 50°C for 10 min. Based on the pH profile results, subsequent experiments were conducted at pH 6.0. For the temperature profile of SCO3486, the enzyme reaction was carried out in the range of 10–80°C. The thermal stability of the enzyme was determined by measuring enzyme activity after heat treatment at the indicated temperatures for 1 h. To confirm the effect of metal ions on enzyme activity, the enzyme activity was measured by adding EDTA or various metal ions (CaCl_2_, CoCl_2_, CuCl_2_, MgCl_2_, MnCl_2_, NiCl_2_, NaCl, ZnCl_2_, KCl, and FeCl_2_) to a final concentration of 5 mM.

### Substrate Specificity of SCO3486

The substrate specificity of SCO3486 was determined under the standard conditions using 1 mM of various substrates (L-AHG, L-glyceraldehyde, D-glyceraldehyde, D-fructose, D-ribose, D-lyxose, D-galactose, D-glucose, L-fucose, and L-rhamnose).

### Identification of the Bioconversion Products of L-AHG by SCO3486 and SCO3480

The products of the SCO3486 and SCO3480 enzyme reactions on L-AHG were determined by thin-layer chromatography (TLC). The enzyme reactions of L-AHG (50 μg) and SCO3486 (15 μg) were carried out under the standard conditions for 12 h.

SCO3480 was expected to be AHGA cycloisomerase converting AHGA to KDGal, and thus sequential reactions on L-AHG by SCO3486 and SCO3480 were carried out. For this purpose, 15 μg of SCO3480 was added to the reaction mixture of SCO3486 described above, and then incubated at 50°C for 12 h.

After heat treatment in boiling water for 5 min, the reaction mixtures were chilled in an ice-water bath for 2 min. The samples were then spotted on a Silica Gel 60 plate and separated with a solvent (n-butanol:ethanol:water = 3:1:1, v/v). The bioconversion products were visualized by spraying with 20% H_2_SO_4_ in methanol and heating at 100°C for 3 min, as previously described [18].

### Mass Spectrometer Analysis of Bioconversion Products

To analyze the molecular masses of the bioconversion products of L-AHG obtained by SCO3486 and SCO3480 catalysis, the unstained region corresponding to the reaction product on the TLC plate was recovered by scraping with a razor blade, and resolved in methanol. Insoluble particles were removed by centrifugation at 15,000 ×g for 10 min, and the supernatant was dried in a CVE-2000 centrifugal evaporator (EYELA, Japan). The dried sample was extracted with methanol and analyzed using liquid chromatography/time-of-flight (LC-TOF) mass spectrometry (JMS-T100LP 4G, JEOL Ltd., Japan). Mass spectra in the *m/z* range 100–250 were obtained using electrospray ionization (ESI) in the positive-ion mode, under the conditions described [5].

## Results

### In Silico Analysis of SCO3486 and SCO3480 from *S. coelicolor* A3(2)

**Fig. 2:**
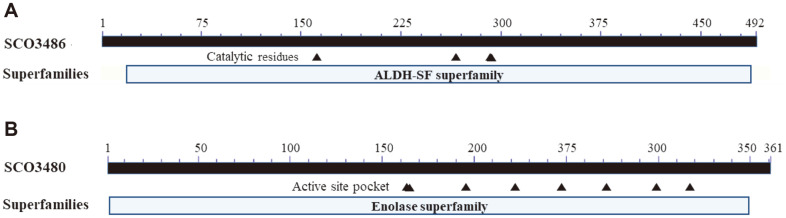
Distribution of conserved domains in SCO3486 and SCO3481. (**A**) The SCO3486 protein (492 amino acids) with NADP^+^-dependent aldehyde dehydrogenase superfamily (ALDH-SF) domain. SCO3486 was annotated as a putative aldehyde dehydrogenase, and had well-conserved catalytic residues (Asn-161, Glu-259, Gly-290, Cys-293) as indicated by filled triangles. (**B**) The SCO3480 (361 amino acids) with mandelate racemase-like subfamily domain of the enolase superfamily. The eight amino acid residues (Lys-164, Lys-166, Asp-195, Glu-221, Glu-247, Asp-270, His-297, and Glu-307) constituting the active site pocket in SCO3480 are depicted by filled triangles.

According to the genomic sequence data [19], the genes for agar degradation, including *dagA* (SCO3471), *dagB* (SCO3487), and *dagC* (SCO3481), are clustered in a 23.6-kb region, which is flanked by transposase genes (SCO3471~SCO3487). Therefore, the genes responsible for the degradation of L-AHG were expected to be present in this cluster. Through in silico analysis, the SCO3486 protein (492 amino acids) was annotated as a putative aldehyde dehydrogenase (ALDH), whose primary structure has 40% identity with that of H2IFE7.1, which encodes the AHG dehydrogenase involved in the initial catabolism of L-AHG to L-AHGA in *Vibrio* sp. EJY3 [9]. SCO3486 has an *Escherichia coli* lactaldehyde dehydrogenase domain (cd07088) with conserved catalytic residues (Asn-161, Glu-259, Gly-290, Cys-293) in the region spanning Phe-19 and Val-488, with an e-value of 0 (Fig. 2A). It belongs to the NADP^+^-dependent aldehyde dehydrogenase superfamily (ALDH-SF), which is known to oxidize a wide range of endogenous and exogenous aliphatic and aromatic aldehydes to their corresponding carboxylic acids, and plays an important role in detoxification [20, 21].

SCO3480 (361 amino acids) has 42% amino acid sequence identity with H2IFX0.1, identified from *Vibrio* sp. EJY3 as an AHGA cycloisomerase that mediates the isomerization of AHGA to KDGal [9]. It has a mandelate racemase (MR)-like subfamily domain (cd03316) in the region covering Ile-2 and Ser-349 with an e-value of 1.46 × e^-123^, and a muconate cycloisomerase domain (TIGR02534) spanning Ile-2 and Leu-358 with an e-value of 6.50 × e^-30^, which belongs to the enolase superfamily. The eight amino acids that make up the active site pocket were also well preserved in the SCO3480 protein (Fig. 2B). The Signal P 4.1 program (http://www.dtu.dk/services/SignalP/) predicted that SCO3486 and SCO3480 had no signal peptide, indicating that they are intracellular proteins [22].

### Heterologous Expression and Purification of the SCO3486 and SCO3480 Proteins

**Fig. 3:**
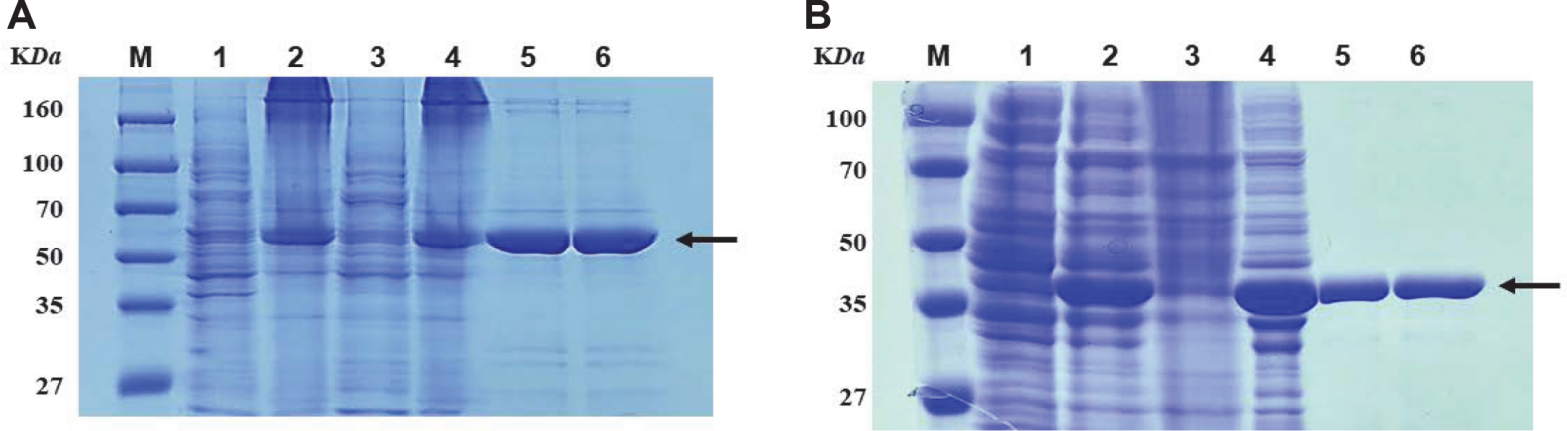
Sodium dodecyl sulfate-polyacrylamide gel electrophoresis (SDS-PAGE) of the purified recombinant proteins SCO3486 (A) and SCO3480 (B). Both recombinant proteins with N-terminus His-tag were purified by TALON metal affinity chromatography from *E. coli* ER2566/pHis-SCO3486 and *E. coli* ER2566/pHis-SCO3480, respectively. Lanes: M, molecular mass marker; 1, cell-free extract before IPTG induction; 2, cell-free extract after IPTG induction; 3, cell debris after centrifugation of IPTG-induced cell lysate; 4, cell-free extract after centrifugation of IPTG-induced cell lysate; 5, purified recombinant proteins; 6, dialyzed recombinant proteins. The migration of the recombinant proteins are indicated by the arrows.

Two recombinant proteins, His-tagged SCO3486 (rSCO3486) and His-tagged SCO3480 (rSCO3480), were successfully overexpressed in *E. coli* ER2566 and purified using TALON metal affinity chromatography. The molecular masses of rSCO3486 and rSCO3480 calculated from their primary sequences were 55,718 Da and 42,924 Da, respectively, which were in good agreement with the molecular weights calculated by SDS-PAGE (Figs. 3A and 3B).

### Enzymatic Properties of rSCO3486

**Fig. 4:**
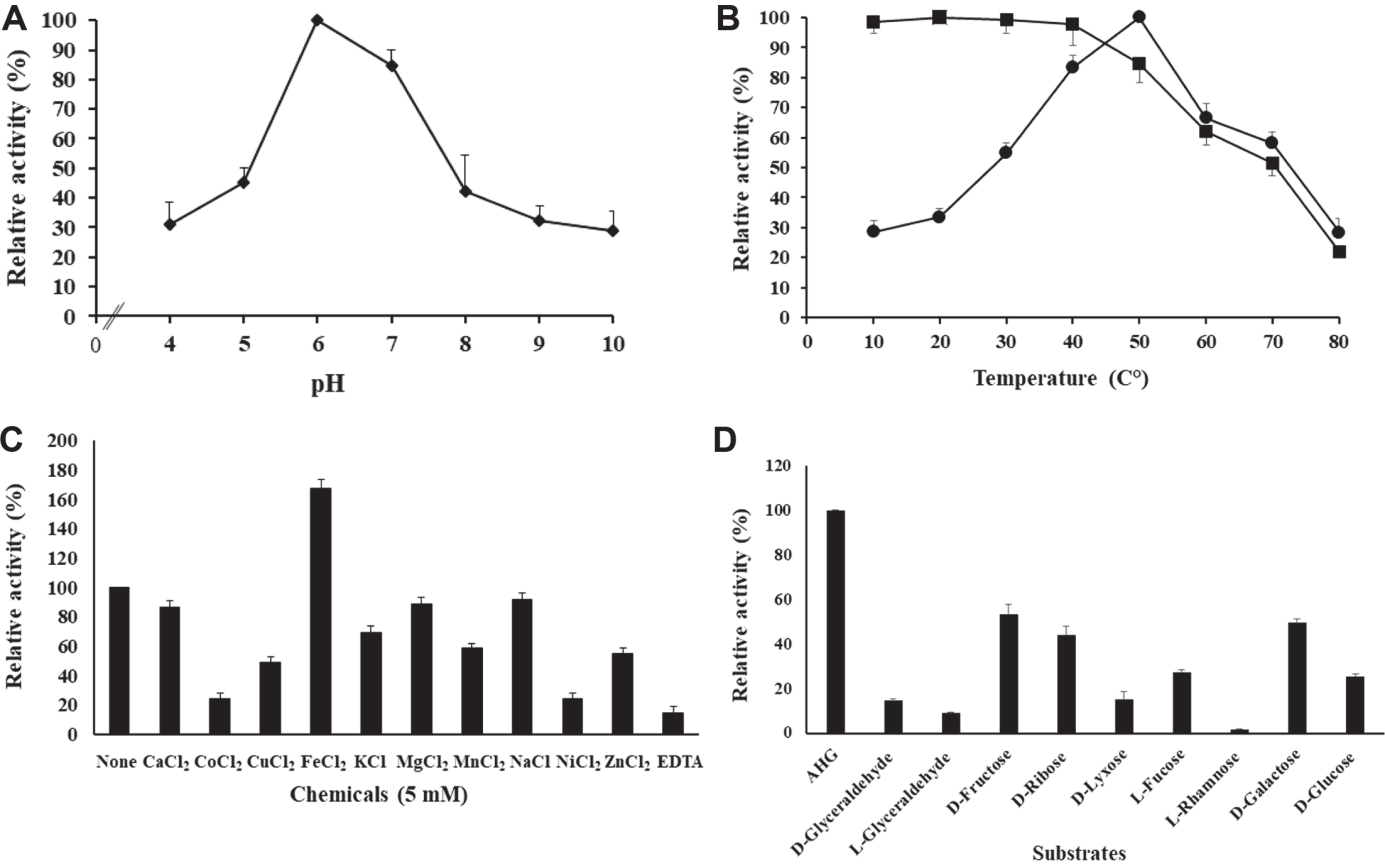
Enzymatic properties of the recombinant SCO3486 protein with respect to 3,6-anhydro-L-galactose. (**A**) Effect of pH: the enzyme activity was measured at 50°C under various pH conditions in 50 mM sodium citrate buffer (pH 4.0 and 5.0), sodium phosphate buffer (pH 6.0 and 7.0), 50 mM Tris-HCl buffer (pH 8.0 and 9.0), and 50 mM glycine-NaOH buffer (pH 10.0). (**B**) Effect of temperature: temperature profile was evaluated at indicated temperatures in 50 mM sodium phosphate buffer (pH 6). The temperature stability was determined at 50°C after pre-incubation at indicated temperatures for 1 h. ●, optimum temperature; ■, thermostability. In (**A**) and (**B**), the highest enzyme activity was set to 100% for calculating the relative activity. (**C**) Effect of metal ions and chelating agent. Enzyme reactions were carried out in the presence of various metal ions and EDTA at final concentration of 5 mM in 50 mM sodium phosphate buffer (pH 6.0) at 50°C. The enzyme activity without chemicals was set to 100%. (**D**) Substrate specificity: enzyme reactions were carried out with various substrates (1 mM) in 50 mM sodium phosphate buffer (pH 6.0) at 50°C. The activity toward L-AHG was considered as 100%. All points are the means of three independent replicates.

Based on in silico analysis, the SCO3486 protein was expected to be an AHG dehydrogenase. To confirm its dehydrogenase activity, rSCO3486 was reacted with L-AHG as substrate and the amount of NADPH generated by the oxidation-reduction reaction coupled with L-AHG oxidation into AHGA was measured. We found that the amount of NADPH increased, which strongly indicated that rSCO3486 mediates the oxidation of L-AHG in a way similar to that of AHG dehydrogenase (Fig. 4).

When the dehydrogenase activity of rSCO3486 toward L-AHG was tested, it was found to be maximum at pH 6.0, while it dropped to 44% and 42% of the maximum at pH 5.0 and 8.0, respectively (Fig. 4A). rSCO3486 showed maximum activity at 50°C, and maintained above 50% of the maximum activity between 40°C and 70°C (Fig. 4B). Thermal stability tests revealed that the enzyme was stable up to 40°C but gradually lost its activity at temperatures above 50°C. EDTA showed a significant inhibitory effect on SCO3486 activity, indicating that rSCO3486 may require a cofactor (Fig. 4C). Although divalent cations such as MnCl_2_, CuCl_2_, ZnCl_2_, NiCl_2_, and CoCl_2_ strongly inhibited enzyme activity, the addition of 5 mM FeCl_2_ remarkably enhanced enzyme activity by 167%.

### Substrate Specificity of rSCO3486

SCO3486 showed maximum activity toward L-AHG among the substrates tested (Fig. 4D). The enzyme activities toward D-fructose, D-galactose, and D-ribose, were between 40% and 50% of the maximum, but those toward L-rhamnose, L-glyceraldehyde, D-glyceraldehyde, L-fucose, and D-glucose were much lower.

### Enzymatic Reaction Product of L-AHG by Sequential Action of SCO3486 and SCO3480

**Fig. 5:**
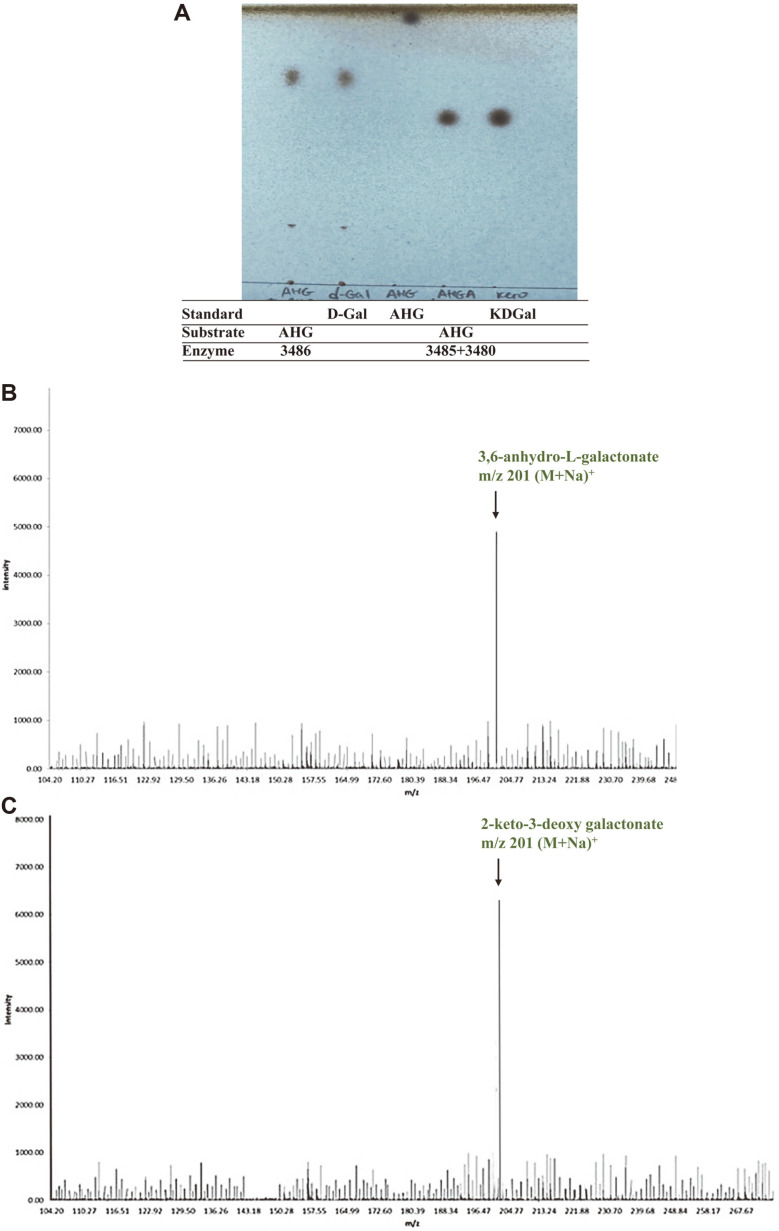
Instrumental analysis of two key L-AHG metabolic intermediates. (**A**) Thin-layer chromatogram: the bioconversion products of L-AHG by SCO3486 only and SCO3486 plus SCO3480 were separated on a silica gel 60 TLC plate. Standards used are 3,6-anhydro-L-galactonate (AHGA), 3,6-anhydro-L-galactose (L-AHG), and 2-keto-3-deoxy galactonate (KDGal). (**B**) LC-TOF mass spectrum of the bioconversion products of L-AHG by SCO3486: the peak for molecular ions at m/ z of 201 corresponding to AHGA is indicated by an arrow. (**C**) LC-TOF mass spectrum of the bioconversion products of L-AHG by SCO3486 and SCO3480: the peak for molecular ions at m/z of 201 corresponding to KDGal is indicated by an arrow.

To investigate the catabolic bioconversion of L-AHG in *S. coelicolor* A3(2), enzymatic reactions were carried out in two sequential steps. In the reaction using rSCO3486 and L-AHG as substrate, a decrease in L-AHG and the appearance of AHGA were observed using TLC, indicating that SCO3486 is an AHG dehydrogenase. In the sequential reaction of SCO3486 and SCO3480 using L-AHG as substrate, a decrease in AHGA and the appearance of KDGal were observed with TLC, indicating that SCO3480 is an AHGA cycloisomerase (Fig. 5A).

The molecular masses of the bioconversion products were analyzed using LC-TOF mass spectrometry (Figs. 5B and 5C). The reaction product of SCO3486 had a molecular mass at *m/z* of 201, corresponding to AHGA (M+Na)^+^. In addition, the reaction product of SCO3486 and SCO3480 had a molecular mass at m/z of 201, corresponding to KDGal (M+Na)^+^.

All these data indicate that L-AHG is oxidized into AHGA by SCO3486 AHG dehydrogenase, and then further converted into KDGal by SCO3480 AHGA cycloisomerase, which is catabolized via the central metabolic pathway in *S. coelicolor* A3(2).

## Discussion

Marine biomass, including red algae, has many advantages as a renewable resource because of its high polysaccharide content and ease of cultivation without the use of pesticides or fertilizers. To maximize the use of a marine biomass such as agarose, it is important to understand the metabolic fate of the monomeric building blocks of agarose, D-Gal and L-AHG, in microorganisms. Although D-Gal metabolism has been validated in detail [23, 24], L-AHG metabolism has not been studied until recently.

The metabolic pathway for L-AHG was first proposed based on data obtained from bioinformatic analysis and wet lab experiments on two agarolytic microorganisms, *Postechiella marina* M091 and *Pseudoalteromonas atlantica* T6c [25]. Based on changes in cofactor concentration caused by enzymatic reactions, the authors suggested that L-AHG may be metabolized to pyruvate and D-glyceraldehyde-3-phosphate via six enzymatic steps. The dehydrogenation reaction converting L-AHG into AHGA by dehydrogenase, and the isomerization reaction converting AHGA into KDGal by cycloisomerase, were proposed for the first two steps.

More concrete experimental data for the L-AHG metabolic pathway were presented based on metabolite and transcriptomic analyses of agarolytic *Vibrio* sp. EJY3 [9]. Through gas chromatography/time-of-flight mass spectrometry and nuclear magnetic resonance, it was confirmed that L-AHG is oxidized to AHGA by an NADP^+^-dependent AHG dehydrogenase (H2IFE7.1 = VejAHGD) and then isomerized to KDGal, an intermediate in the DeLey–Doudoroff pathway of oxidative galactose metabolism [23, 24], by an AHGA cycloisomerase (H2IFX0.1 = VejACI). The introduction of these two genes into an ethanologenic *E. coli* strain conferred the ability to grow on L-AHG as a sole carbon source and increased ethanol production in the strain, suggesting that the two enzymes are essential for the metabolism of L-AHG [9]. However, all three agar-degrading bacteria that have been studied so far are marine microorganisms belonging to the gram-negative category.

We wondered what the L-AHG metabolic pathway would be in the gram-positive soil microorganism *S. coelicolor* A3(2). In the present study, we identified the two enzymes SCO3486 and SCO3480, which are involved in the L-AHG catabolic pathway as AHG dehydrogenase and AHGA cycloisomerase, respectively, in *S. coelicolor* A3(2). Therefore, the L-AHG catabolic pathway was exactly the same as that mediated successively by VejAHGD and VejACI in *Vibrio* sp. EJY3. All these results imply that the metabolic pathway of L-AHG, a rare sugar, may be the same for both gram-positive and gram-negative agarolytic bacteria.

The SCO3486 AHG dehydrogenase belongs to the NADP^+^-dependent ALDH superfamily, which is involved in various biological processes, especially decreasing oxidative stress caused by aldehydes [20, 21]. Most ALDHs have evolved to have broad substrate specificities toward various aldehyde substrates, such as acetaldehyde, glyceraldehyde, and glycolaldehyde, which is expected, considering that ALDH is responsible for intracellular detoxification of aldehyde compounds [26]. Similarly, SCO3486 showed broad substrate specificities, including D-fructose, D-galactose, and D-ribose, but displayed the highest activity toward L-AHG.

In contrast, VejAHGD from *Vibrio* sp. EJY3 showed high substrate specificity for L-AHG alone. It did not show any catalytic activity toward other aldehyde sugars, including the D-forms of AHG, glyceraldehyde, glucose, galactose, fructose, ribose, and lyxose, and L-forms of glyceraldehyde, lactaldehyde, rhamnose, and fucose [27]. The optimal conditions for enzyme activity were pH 6.0 and a temperature of 50°C in SCO3486, and pH 7.0 and a temperature of 30°C in VejAHGD. The enzyme activity was enhanced by iron ions in SCO3486, and by manganese ions in VejAHGD [9]. These results indicate that the biochemical properties of SCO3486 and VejAHGD are quite different and may be attributed to the low amino acid sequence similarity between the two enzymes.

The NADP^+^-dependent ALDH superfamily forms an oxyanion thiohemiacetal intermediate between the Cys residue and aldehyde substrates, thereby transferring hydride to the cofactor while forming the thioacylenzyme intermediate. Therefore, the role of Cys-293 in the SCO3486 protein is expected to be important among the four catalytic residues. Moreover, recent studies on three-dimensional structures of AHG dehydrogenase revealed that Glu-259 and Cys-293 are important for its catalytic activity [28].

AHGA cycloisomerases, including SCO3480, belong to the enolase superfamily [29] and catalyze a reaction similar to that of galactarolactone cycloisomerase in the galacturonate metabolic pathway [30]. Interestingly, SCO3480 showed 99% and 98% amino acid sequence identities with the two proteins annotated as mandelate racemase/muconate lactonizing enzyme family protein, WP_103546198.1 from *Streptomyces* sp. SM1, and WP_164248662.1 from *Streptomyces* sp. S4.7, respectively. Except for these two proteins, SCO3480 showed low identity with orthologs from other genera. Thus, it is highly likely that the two proteins from *Streptomyces* are AHG dehydrogenases, and *Streptomyces* sp. SM1 and *Streptomyces* sp. S4.7 may be able to utilize agar for growth, which requires further verification.

In conclusion, we validated the metabolic pathway of L-AHG for the first time in the gram-positive bacterium *S. coelicolor* A3(2). We expect that the L-AHG metabolic pathway will provide a useful platform for the efficient production of industrial chemicals and biofuels from red macroalgal biomass.
